# Aging‐related histone modification changes in brain function

**DOI:** 10.1002/ibra.12106

**Published:** 2023-05-17

**Authors:** Yanwen Ding, Chengxi Liu, Yi Zhang

**Affiliations:** ^1^ Department of Anesthesiology The Second Affiliated Hospital of Zunyi Medical University Zunyi Guizhou China; ^2^ Guizhou Key Laboratory of Anesthesia and Organ Protection Zunyi Medical University Zunyi Guizhou China; ^3^ School of Anesthesiology Zunyi Medical University Zunyi Guizhou China

**Keywords:** acetylation, aging, cognitive dysfunction, methylation, neurodegeneration

## Abstract

Aging can be defined as a decline of physiological function that is more difficult to reverse, characterized by the loss of the physiological integrity of tissues, organs, and cells of an organism over time. Normal aging is associated with structural and functional changes in the brain, involving neuronal apoptosis, synaptic structure, neurotransmission, and metabolism alterations, leading to impairment in sleep, cognitive functions, memory, learning, and motor and sensory systems. Histone modification is a significant aging‐related epigenetic change that influences synaptic and mitochondrial function and immune and stress responses in the brain. This review discusses the changes in histone modifications that occur during brain aging, specifically methylation and acetylation, and the associated changes in gene transcription and protein expression. We observed that genes related to synaptic and mitochondrial function are downregulated in the aging brain, while genes related to immune response and inflammatory functions are upregulated.

## Graphical abstract

Alterations in histone modifications play a key role in the loss of partial brain function due to aging. Decreased synaptic and mitochondrial function is a distinctive feature of aging, and these functionally related genes are often accompanied by a decrease in transcriptional activation modification and an increase in inhibitory histone modification during aging, which ultimately leads to a decrease in protein expression. Increased stress and immune responses are often observed during aging, resulting in increased protein expression due to opposite histone modifications in the genes associated with them. This review examines the changes in histone modifications, including methylation and acetylation, that take place as the brain ages and emphasizes the alterations in gene transcription and protein production that these modifications bring about throughout these functional decreases.

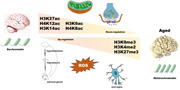

## 1 INTRODUCTION

Aging is a progressive functional deterioration of an organism at the molecular, cellular, and physiological levels. Aging is a major risk factor for numerous diseases, including cancer, cardiovascular diseases, and neurological disorders.^1^ The brain is the organ more likely affected by aging, and cognitive impairment may worsen over time as patients become older. Numerous epigenetic alterations, such as DNA methylation and histone modifications, have been identified in the aging brain as regulators of gene expression associated with learning and memory, synaptic structure development, and synaptic plasticity. This establishes the molecular basis for cognitive decline in the aging brain.

A histone modification is a covalent posttranslational alteration of histone proteins catalyzed by chromatin‐modifying enzymes that dynamically add or remove particular histone residues. Histone modifications can affect chromatin structure by altering the DNA–histone and histone–histone interactions in the nucleosome.^2^ Several studies report a significant contribution of histone acetylation and methylation to brain aging. Here, we review the function and mechanism of histone modifications in brain aging.

## 2 HISTONE ACETYLATION AND METHYLATION MODIFICATIONS

Posttranslational modifications of histones, such as methylation, acetylation, and ubiquitination, can regulate gene expression. These changes have the potential to restructure chromatin and alter expression levels.

**Figure 1:**
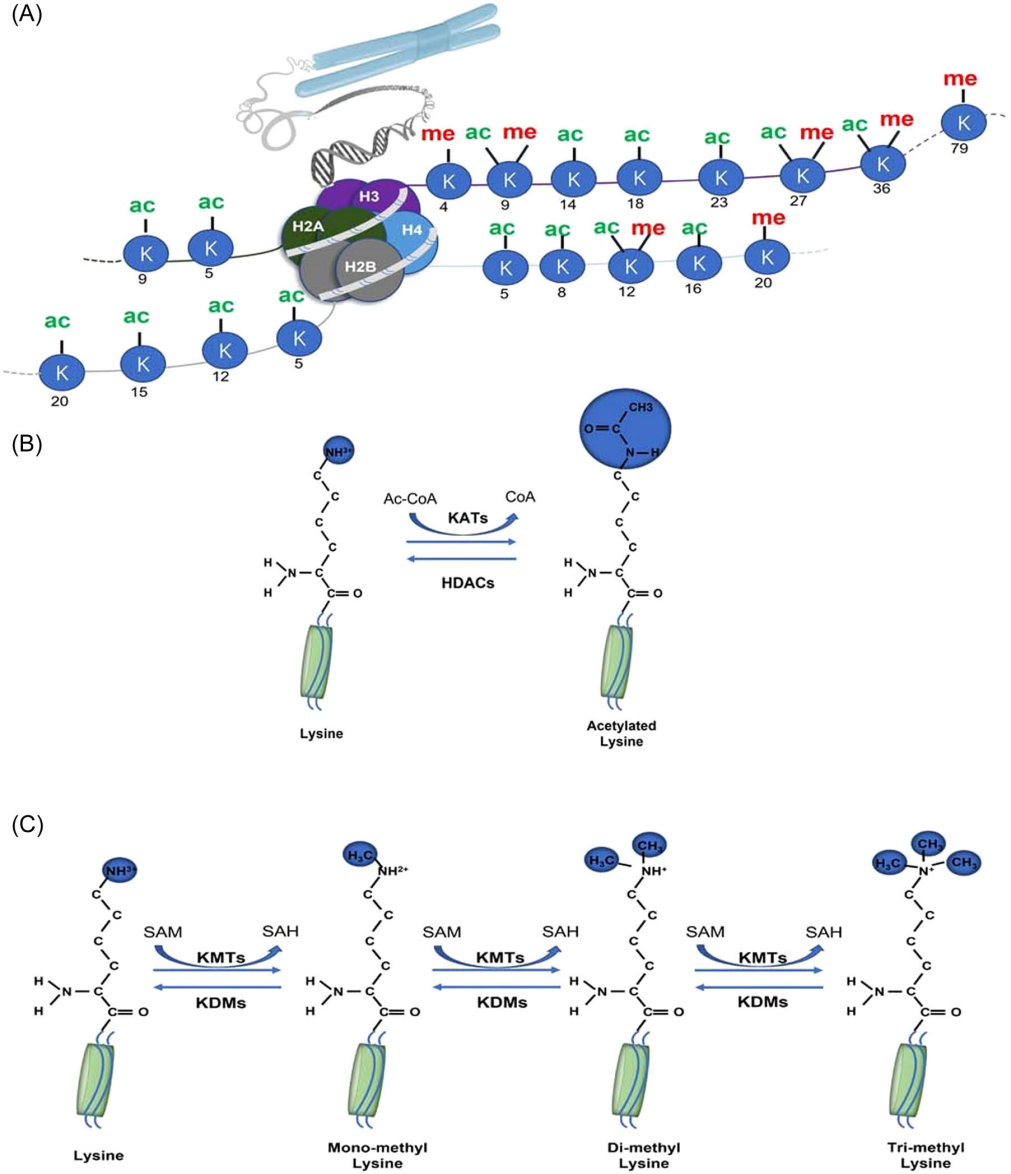
Schematic of histone acetylation and the methylation modification. (A) Schematic representation of the nucleosome showing principal lysine acetylation and methylation sites on histones H3 and H4. ac stands for acetylation; me stands for methylation; and K stands for lysine. (B) K‐acetyltransferases (KATs) transfer acetyl groups from acetyl‐coA to lysine residues of histones. In contrast, histone deacetylases (HDACs) remove acetyl groups from acetylated lysines. (C) K‐methyltransferases (KMTs) transfer methyl groups from S‐adenosylmethionine (SAM) to lysine residues of histones to produce S‐adenosylhomocysteine (SAH). KMTs catalyzes the addition of one, two, or three methyl groups of lysine residues in histones, resulting in monomethylation, dimethylation, or trimethylation of histones, respectively. In contrast, K‐demethylases remove methyl from methylated lysine. [Color figure can be viewed at wileyonlinelibrary.com]

Histone acetylation and deacetylation are highly reversible and tightly regulated modifications that are mediated through K‐acetyltransferases (KATs) and histone deacetylases (HDACs), respectively (Figure 1A,B).^3^ KATs are grouped into three major families based on the structural and functional characteristics of their catalytic centers: (1) the GNAT (Gcn5‐related N‐acetylase) family; (2) the MYST family, consisting of Tip60, MOZ/MYST3, MORF/MYST4, HBO1/MYST2, and hMOF/MYST1; and (3) the p300/CBP (CREB‐binding protein) family, including p300 and CBP.^4^ KATs use acetyl CoA as a cofactor to acetylate lysine amino groups, which neutralizes the positively charged lysine and weakens the interaction between histones and DNA.^5^ This effect loosens the chromatin structure and promotes DNA double‐helix unraveling, increasing DNA accessibility to transcription factors and activating gene transcription.^6^ HDACs remove the acetyl group from ε‐N‐acetyl lysine residues of histone proteins. Based on phylogenetic comparison with yeast homologs, 18 HDACs have been identified in higher mammals and categorized into classes I, II, III, and IV.^7^ Class I includes HDAC1, 2, 3, and 8, which are expressed ubiquitously and localized mainly in the nucleus. Class II HDACs are further subdivided into two subgroups, with Class IIa consisting of HDAC4, 5, 7, and 9, whereas HDAC6 and 10 belong to Class IIb. Class II HDACs are not only found in the nucleus but they are also expressed more selectively. Sirtuin, a Class III HDAC, is found in the cytoplasm, nucleus, and mitochondria. They have a similar sequence to yeast Sir2 and are made up of SIRT1–7. HDAC11 is the only member of the Class IV HDAC family that is primarily found in the nucleus.^8^ Histone acetylation is correlated with increased transcriptional activity, whereas histone deacetylation is associated with transcriptional repression.

Histone methylation is defined as a common histone mark by adding a methyl group (‐CH3) onto the lysine or arginine residues of histones.^9^ (Figure 1A,C) Histone–lysine *N‐*methyltransferases are histone‐modifying enzymes that catalyze the transfer of one, two, or three methyl groups to lysine residues of histone proteins.^10^ Meanwhile, the arginine *N*‐methyltransferase undergoes only one or two methylations (symmetric or asymmetric).^11^ Various levels of methylation significantly increase the complexity of histone methylation modification and gene expression regulation.^9^, ^12^ Histone methylation is catalyzed by a class of enzymes, Histone methyltransferases (HMTs), which can reversibly methylate specific residues of histones. According to the amino acids catalyzed by this enzyme, HMTs are divided into two families: K‐methyltransferases (KMTs) and protein arginine methyltransferases (PRMTs).^13^ K‐demethylases (KDMs) remove methyl groups from lysine or arginine residues of histone proteins. Two major families of evolutionarily conserved KDMs have been identified: lysine‐specific demethylases (LSD) and JmjC family N‐methyl lysine demethylases (JMJC), which use different reaction mechanisms to establish demethylation.^14^

## 3 THE ROLE OF HISTONE ACETYLATION CHANGES AND THE UNDERLYING MECHANISMS IN BRAIN AGING

### 3.1 Histone acetylation changes associated with synaptic function

In the human prefrontal cortex, *GATA3* and other genes associated with neuronal and synaptic function show age‐dependent downregulation along with reduced histone 3 lysine 27 (H3K27ac) acetylation at their promoters. Cheng et al.^15^ have reported similar findings in aged mice, and treatment of histone deacetylase inhibitor Vorinostat (SAHA) attenuated age‐related neuronal and synaptic dysfunction. Aging is reported to decrease H4K12ac at the intron–exon junction of genes involved in synaptic function in the mouse hippocampal region, disrupting their selective splicing and resulting in abnormal gene expression and impaired memory. Surprisingly, after the administration of SAHA, these modifications were almost completely restored.^16^

The levels of mRNA for brain‐derived neurotrophic factor (BDNF) exon II or VI were significantly reduced in the hippocampus of aged mice compared to adult mice. Additionally, activation marker H3K27ac was considerably reduced on the promoters of these exons in the hippocampal region of aged mice.^17^ The Palomenr team also found that the CREB mediates H3K27 acetylation by recruiting KATs CBP to these promoters.^18^ CBP levels on these two promoters in the aged hippocampus are also markedly decreased.^17^ Hippocampal aging is accompanied by an increase in HDAC2 at *Bdnf* exons II and VI. HDAC2 is known to regulate memory formation.^17^ Overall, the decrease of KATs CBP and the increase of deacetylase HDAC2 in the hippocampus of older mice combined to decrease H3K27ac, resulting in downregulation of the expression of *Bdnf* exons II and VI.

Overall, HDAC2 levels are reported to be elevated in the hippocampus of aged mice.^19^ Similarly, the binding of HDAC2 to the promoter of neuronal immediate early genes (IEGs) increases with age.^20^ IEGs are cells' first set of genes to be expressed within an hour of external stimulation without the need for de novo protein synthesis.^21^ IEG expression is widely used as a molecular marker of long‐term memory and synaptic plasticity.^22^, ^23^ Increased HDAC2 activity reduces the expression of neuronal IEGs by decreasing the acetylation of H3K9 and H3K14 at the promoters of these genes.^20^ In contrast, inhibition of HDACs increased synaptic plasticity, increased the number of dendritic spines, and improved memory in young animals.^24^ Specific inhibition of HDAC2 enhanced the acetylation of H3K9 and H3K14 at neuronal IEG promoters, restored their expression in the hippocampus of aged mice, and prevented memory impairment.^20^

HDAC3 is a significant contributor to the decline in learning and memory. Elevated H4K8ac in the hippocampus and nucleus accumbens of HDAC3‐FLOX mice led to a significant increase in the expression of Nuclear receptor subfamily 4 group A member 2 (Nr4a2) and c‐fos, thereby improving age‐related impairments in long‐term memory and synaptic plasticity.^25^, ^26^, ^27^ In addition, deleting HDAC3 in the mouse model reversed age‐related deficits in the hippocampal long‐term potentiation (LTP).^28^

Overall, during brain aging, a decrease in histone acetyltransferase, or an increase in histone deacetylation, leads to a decrease in some acetylation markers such as H3K27ac, H4K12ac, H3K27ac, H3K9ac, and H3K14ac, inducing a series of reductions in the expression of key genes to neuronal and synaptic development, ultimately manifesting as a decrease in age‐related memory learning capacity.

### 3.2 Histone acetylation associated with mitochondrial function

An imbalance in the NAD+/NADH ratio affects the activity of NAD+‐dependent HDAC in aging neurons.^29^ With age, the activity of NAD+‐dependent deacetylases known as sirtuins steadily decreases as NAD+ levels decline in cells.^29^, ^30^ SIRT1 can regulate axonal growth and synaptic processes involved in cognitive function and synaptic plasticity.^31^, ^32^ Decreased SIRT1 activity in aging neurons may impair cognitive function in the elderly. *Sirt1* knockout hippocampal CA1 neurons had lower levels of synaptophysin, impaired LTP, and lower dendritic density.^32^ CREB levels are reduced in *Sirt1*‐deficient mice, which impairs the binding of CREB to BDNF and may result in reduced BDNF levels in the brain.^33^ SIRT1 modulates synapse formation and synaptic plasticity to regulate memory formation. Mitochondria provide key metabolites such as NAD+, ATP, and acetyl‐CoA, which are required for many transcriptional and epigenetic processes. The age‐related decline in mitochondrial function leads to a decrease in the activity of NAD+‐dependent deacetylases sirtuins, ultimately manifesting in the reduced ability of SIRT1 to regulate synaptic formation and synaptic plasticity to regulate memory formation.

### 3.3 Histone acetylation associated with stress and inflammatory immune response

Aging results in an increase in stress sensitivity.^34^ Age‐related environmental stress can lead to cognitive decline and neurodegeneration through hyperactivation of the hypothalamic–pituitary–adrenal axis and elevated glucocorticoid levels. Sustained elevation of corticosterone induced by chronic mild stress can specifically downregulate CREB activity and BDNF expression in the dentate gyrus.^35^ Reduced CREB activity reduces its ability to recruit KATs and KDMs and regulate gene expression. This has been shown to reduce hippocampus synaptic plasticity and increase the risk of neuronal degeneration.^36^, ^37^

An integrative study of H3K27ac profiling and gene expression in human and mouse brains by Cheng et al.^15^ found that age‐related upregulated and downregulated genes show distinct H3K27ac patterns. This type of acetylation is distributed on the promoters of both types of genes, but genes with inflammatory immune‐related functions, such as the nuclear factor‐κB (NF‐κB) and B‐cell lymphoma‐3 (BCL3), have more extensive H3K27ac alterations on their gene bodies and are progressively reduced with aging. Hyperacetylation of the gene body suppresses the hyperactivation of associated genes, which are gradually lost with aging; as a result, inflammatory senescence‐related genes are activated, in contrast to the promoter region H3K27ac's transcriptional activation function.^15^ Hippocampal neuroinflammation is common in elderly patients with increased HDAC expression and postoperative cognitive impairment. Treatment of SAHA or UF010, a selective inhibitor of class I HDAC, significantly reduces hippocampal neuroinflammation and improves postoperative cognitive dysfunction by inactivating NF‐κB, Janus kinase/signal transducers and activators of transcription (JAK/STAT), and Toll‐like receptor/myeloid differentiation primary response protein (TLR/MyD88) signaling pathways.^38^

Aging causes increased stress, downregulates the expression of CREB, and decreases binding to histone acetylatinase, promoting the degeneration of neurons. Aging also promotes the increase of H3K27ac on the gene of many inflammatory immune‐related functions, which increases the inflammatory activation of the brain with age and promotes cognitive impairment. This suggests that partial histone acetylation changes promote the activation of stress and immunoinflammation during aging, leading to brain dysfunction.

**Figure 2:**
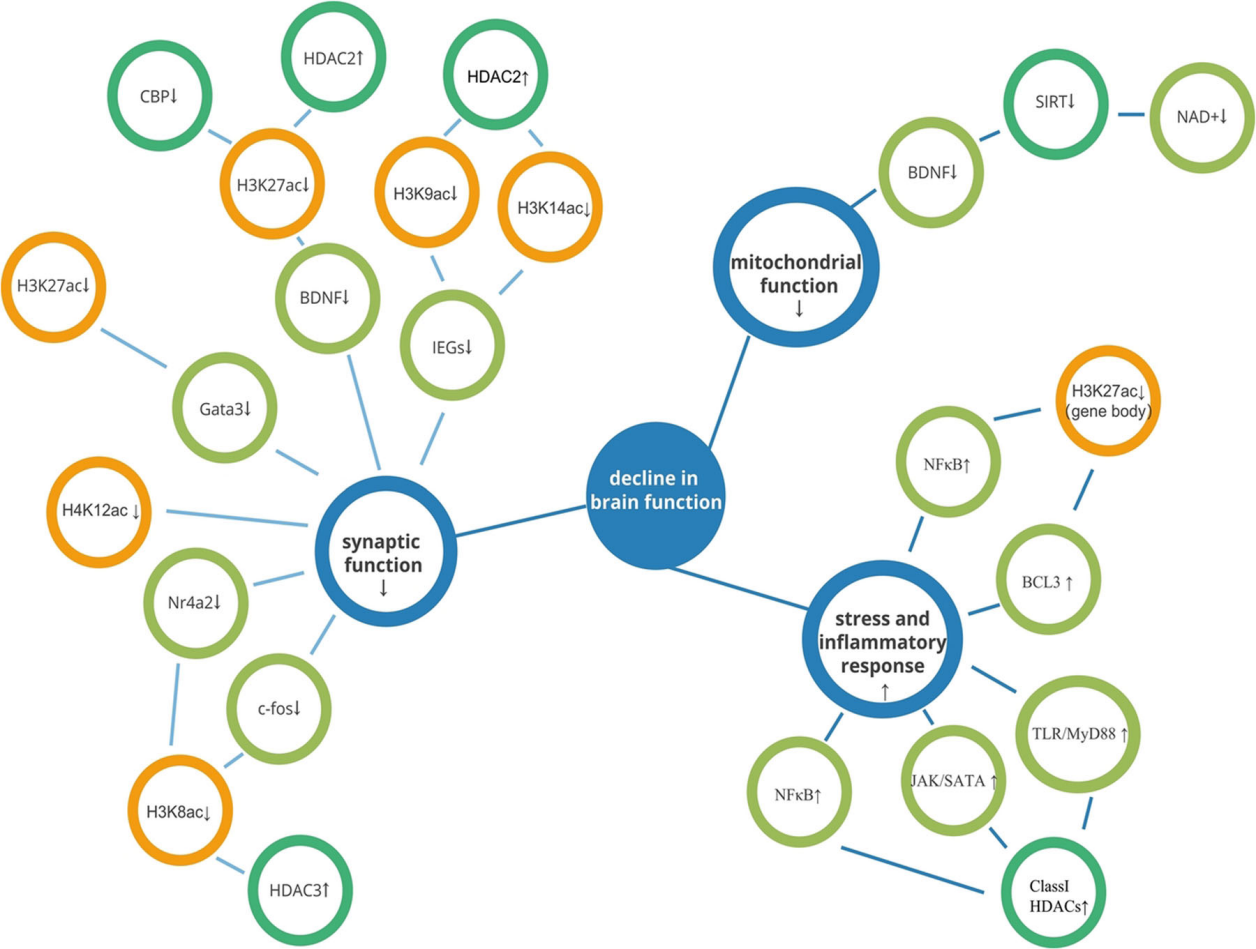
Partial mechanism of histone acetylation changes in the aging brain. The changes of histone acetylation related to aging are involved in synaptic function, mitochondrial function, stress, and inflammatory response, and ultimately lead to the decline of brain function. [Color figure can be viewed at wileyonlinelibrary.com]

Histone acetylation is seen as a sign of euchromatin since it is typically linked to transcriptional activation through the recruitment of transcription factors and RNA polymerase Ⅱ.^39^ As a result, decreased expression of related genes is frequently seen with aging due to loss of histone acetylation. Yet, there are also other patterns (Figure 2, Table 1).

**Table 1:** Epigenetic determinants of altered functional gene expression in the aging brain.

Localization	Histone modification	Function
Changes in the expression of synapse‐related functional genes in the aging brain		
Prefrontal cortex	H3K27ac↓	GATA3↓
Hippocampus	H4K12ac↓	——
	H3K27ac↓	BDNF↓
	H3K9ac↓,H3K14ac↓	IEGs↓
	H4K8ac↓	Nr4a2↓,c‐fos↓
	H3K9me3↑	BDNF↓, GluR1↓, IEGs↓
Changes in the expression of mitochondria‐related functional genes in the aging brain		
Aging neuron	SIRT1↓, CREB↓	BDNF↓
Prefrontal cortex	EHMT1↑, H3K9me3↑	——
Changes in stress response and expression of immune‐related functional genes in the aging brain		
Aging neuron	CREB↓	——
Brain	H3K27ac(gene body)↓	NF‐κB↑, BCL3↑
Hippocampus	HDACs↑	NF‐κB↑, TAK/SATA↑, TLR/MyD88↑
Prefrontal cortex	H3K4me2↑, SETD7↑, DPY30↑	UBXN4↑, TUFT1↑, SLC24A4↑
Microglia	H3K27me3↑, Jmjd3↓	Arginase‐1↑, CD206↑

*Note*: Related to synaptic and mitochondrial function are downregulated in the aging brain, while genes related to immune response and inflammatory functions are upregulated.

Abbreviation: BDNF, brain‐derived neurotrophic factor.

## 4 THE ROLE AND MECHANISM OF HISTONE METHYLATION ALTERATIONS IN THE AGING BRAIN

### 4.1 Synaptic function‐related histone methylation alterations

BDNF is a molecule that regulates synaptic plasticity in the hippocampus. In the hippocampus of aged mice, inhibitory histone H3 trimethylation of histone 3 lysine 9 (H3K9me3) was reported to be increased at the BDNF promoter.^40^ SUV39H1 (H3K9 KMTs) inhibition reduced H3K9me3 levels in the hippocampus of aged mice. SUV39H1 inhibition promoted dendritic growth and stability while increasing the surface level of glutamate receptor 1 in hippocampal synaptosomes, restoring hippocampal memory.^40^

IEGs, a molecular marker of synaptic plasticity and long‐term memory, contribute to signal transduction during memory consolidation.^23^, ^41^ With age, IEGs such as activity‐regulated cytoskeletal (Arc), early growth response 1 (Egr1), homer scaffold protein 1 (Homer1), and neuronal activity‐regulated pentraxin (Narp), are significantly downregulated,^20^ which results in decreased learning and memory abilities. Kushwaha and colleagues found that the underlying mechanism may be associated with increased expression of H3K9me3 in the promoter region of neuronal IEGs, which results in heterochromatinization and downregulation of the corresponding protein expression. The primary KMTs SUV39H1, which is responsible for the age‐related increase in H3K9me3, is markedly elevated in the hippocampus of aged mice.^42^ Targeted inhibition of SUV39H1 decreased H3K9me3 levels and reversed the age‐related deficits in hippocampal memory function.^40^

Aging causes an increase in H3K9me3 in the gene promoter region of synaptic plasticity regulatory important molecules such as BDNF and IEGs, and the expression of corresponding proteins is downregulated, resulting in memory impairment. Moreover, the use of methyltransferase inhibitors can improve synaptic plasticity and stability, and restore memory function.

### 4.2 Histone methylation changes associated with mitochondrial function

In the human prefrontal cortex, there is an age‐related increase in the expression levels of H3K9 methyltransferase euchromatic histone methyltransferase 1 (EHMT1) and epigenetic‐related recognition factor, the bromodomain adjacent to the zinc finger 2B gene (BAZ2B). It is, however, more pronounced in Alzheimer's disease (AD) patients' brains and is positively correlated with the progression of AD.^43^ In nematodes, homologous proteins called SET‐6 and BAZ‐2 can regulate H3K9me3 methylation modifications in the promoter regions of mitochondria‐related genes, preventing their expression. In contrast, knockout of *Set‐6* and *Baz‐2* significantly increased neurotransmitter levels in aged nematodes and delayed age‐related behavioral deficits. Similarly, knockout of the homologous gene *Baz2b* in mice delayed aging‐related decline in cognitive behavior.^43^ Additionally, increased expression levels of EHMT1 have been confirmed in both the AD mouse model and human AD brain tissue. Pharmacological inhibition of elevated EHMT1 could maintain proper mitochondrial function and enhance learning and memory in the AD mice model.^44^ This indicates that the increase of H3K9 methyltransferase can be observed not only in aging individuals but also in AD, a neurological disease highly associated with age, leading to an increase in the level of H3K9me3 in the promoter region of mitochondrial function‐related genes, which is manifested as a decline in learning and memory ability caused by the impairment of mitochondria‐related functions.

### 4.3 Histone methylation associated with inflammatory immune and stress response

Age‐mediated accumulation of H3K4me2 at promoters of several stress response‐related genes is reported in the monkey brain prefrontal cortex, which, by activating transcription, increased the expression of the stress response‐related proteins UBX domain protein 4 (UBXN4), tuftelin 1 (TUFT1), and Solute carrier family 24, member 4 (SLC24A4), leading to cognitive decline.^45^ A similar study found that the expression of two H3K4 methyltransferases, the SET domain containing lysine methyltransferase 7 (SETD7) and Dumpy‐30 (DPY30), increased with age. This finding implies that the increased stress response mediated by H3K4me2 could be due to increased methylation rather than decreased demethylation.^45^

Age‐related alterations in the immune system are accompanied by microglial dysfunction. Senescent microglia can no longer establish effective immune responses and maintain normal synaptic activity, which directly contributes to age‐related cognitive deficits and neurodegeneration.^46^ A study reported reduced levels of the histone H3K27me3 demethylase Jumonji Domain‐Containing Protein 3 (Jmjd3) in the midbrain of aged mice, which is a necessary enzyme for microglia activation. This is accompanied by an increase in the level of H3K27me3, which promotes a switch in the microglia phenotype from M2 to M1, thereby exacerbating microglia‐mediated inflammatory responses.^47^ The importance of Jmjd3 in M2 microglia polarization is demonstrated by increased H3K27me3 levels and decreased expression of M2 markers such as arginase 1 and mannose receptor (MR or CD206) in *Jmjd3* knockout microglia.^47^

Aging can increase the expression of H3K4 methyltransferase and trigger the accumulation of H3K4me2 in the promoter region of various stress‐related genes, resulting in increased expression of related stress‐response proteins and inducing cognitive impairment. At the same time, aging reduces the histone demethylase Jmjd3, resulting in increased levels of H3K27me3 and exacerbating inflammation. It is suggested that histone methylation may cause neurodegeneration and dysfunction by activating the stress and immune inflammation in the aging process.

**Figure 3:**
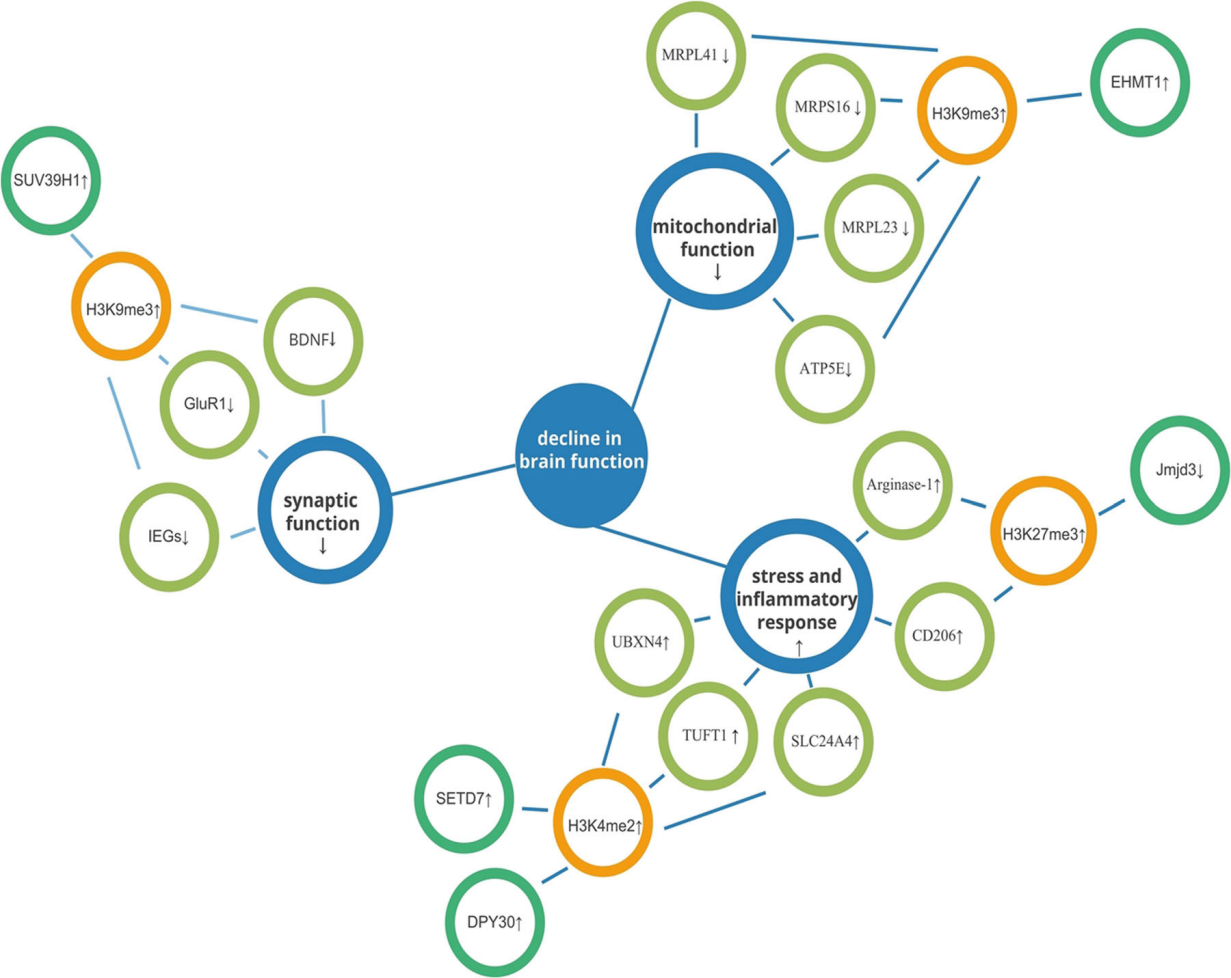
Partial mechanism of histone methylation changes in the aging brain. The changes of histone methylation related to aging are involved in synaptic function, mitochondrial function, stress, and inflammatory response, and ultimately lead to the decline of brain function. [Color figure can be viewed at wileyonlinelibrary.com]

Histone methylation, unlike histone acetylation, can result in either transcriptional activation or repression depending on the pattern of methylation. Specifically, the amount of methyl supplied and the changed histone location determine the direction of transcriptional control.^39^ Thus, the alterations in histone methylation observed during aging are more complex, with associated genes undergoing upregulation or downregulation as methylation changes (Figure 3, Table 1).

## 5 CONCLUSION

In the aging brain, epigenetic modifications that promote gene expression are reduced, whereas epigenetic modifications that inhibit gene expression are increased in the promoter regions of genes related to neuronal function, synaptic transmission, and learning and memory. Therefore, the expression of these target genes declines with age, leading to cognitive impairment. The opposite is true for genes associated with immune response and inflammatory function. Further research is needed to complete the histone modification landscape of brain aging and understand how aging‐mediated changes in histone modifications influence cognitive and behavioral decline. Because these changes are reversible, the pharmacological intervention of histone modification status can be considered a novel strategy in treating age‐related cognitive and behavioral decline.

## AUTHOR CONTRIBUTIONS

Yanwen Ding and Yi Zhang contributed to the main conception and resource collection. Chengxi Liu contributed to drafting and editing; and Yi Zhang finalized the review and approved the final version.

## CONFLICT OF INTEREST STATEMENT

The authors declare no conflict of interest.

## ETHICS STATEMENT

The ethics statement is not available.

## Data Availability

The data is widely open and available.
